# Rectal Hypersensitivity in Inflammatory Bowel Disease: A Systematic Review and Meta-analysis

**DOI:** 10.1093/crocol/otab041

**Published:** 2021-06-23

**Authors:** Christopher Roberts, Ahmed Albusoda, Adam D Farmer, Qasim Aziz

**Affiliations:** 1 Centre for Neuroscience, Surgery and Trauma, Blizard Institute, Wingate Institute of Neurogastroenterology, Barts and the London School of Medicine and Dentistry, Queen Mary University of London, London, UK; 2 University Hospital Southampton, Southampton, UK; 3 Mech-Sense, Department of Gastroenterology, Aalborg University Hospital, Aalborg, Denmark; 4 Institute of Applied Clinical Sciences, University of Keele, Keele, UK; 5 Department of Gastroenterology, University Hospitals of North Midlands NHS Trust, Stoke on Trent, UK

**Keywords:** inflammatory bowel disease, abdominal pain, rectal hypersensitivity, IBD–IBS crossover

## Abstract

Abdominal pain is a key symptom of inflammatory bowel disease (IBD), particularly in active IBD, but also occurs in patients with quiescent disease suggesting that mechanisms other than active inflammation may be responsible. Putative hypothesis to explain chronic abdominal pain in patients with quiescent IBD includes crossover with irritable bowel syndrome where rectal hypersensitivity is common and has pathophysiological implications. In contrast, in IBD, the role of rectal hypersensitivity has not been established. We aimed to determine if rectal hypersensitivity was more common in IBD compared to a healthy control population. We searched MEDLINE and EMBASE databases (1970–2018). Prospective studies that measured pain/discomfort thresholds to mechanical rectal stimuli in IBD and healthy controls were included. Data were pooled for meta-analysis and effect sizes were calculated with 95% confidence intervals (CIs). Our search strategy identified 222 citations of which 8 met the inclusion criteria, covering 133 individuals with IBD (67 men), aged between 10 and 77 compared to 99 healthy controls (55 men), aged between 10 and 67. The prevalence of rectal hypersensitivity in IBD compared to healthy controls was similar with an effect size of 0.59 (95% CIs: −0.27 to 1.44, *P* = .16, *I*^2^ = 87.3%). Subgroup analysis did show a significant effect size for patients compared to healthy controls with active disease (1.32) but not for quiescent disease (−0.02). These results suggest that reduced rectal pain thresholds to experimental stimulation are not seen in IBD populations except during active flares of the disease. Further research is required to understand the pathophysiology of chronic abdominal pain in quiescent IBD populations with and without chronic abdominal pain to identify appropriate management strategies.

## Introduction

Rectal hypersensitivity is defined as increased sensitivity to experimental stimuli applied to the gastrointestinal (GI) tract.^1^ It can arise due to a combination of either heightened sensitivity to noxious stimuli (hyperalgesia) and/or non-noxious stimuli (allodynia) due to factors such as peripheral and central sensitization.^2^ Additional mechanisms include alterations in central factors such as aberrant brain processing^3^ and abnormal descending inhibitory control of pain pathways.^4, 5^

Inflammatory bowel disease (IBD) is characterized by chronic noninfectious inflammation of the GI tract, the 2 most common subtypes being ulcerative colitis (UC) and Crohn’s disease (CD). IBD affects 6.8 million individuals worldwide and its age-standardized prevalence rates are increasing over time putatively as a result of urbanization, increasingly hygienic environments, increased meat consumption, and a lower intake of dietary fiber.^6^ The natural history of IBD includes periods of symptomatic flares with periods of remission. Abdominal pain is a key feature of symptomatic flares of disease where there is significant inflammation. Therefore, one of the main treatment goals is to heal inflammation and achieve a symptom-free remission. However, chronic abdominal pain remains a prominent feature in a significant proportion of patients with quiescent disease.

Around 39% of patients with IBD have irritable bowel syndrome (IBS)-type symptoms, this has been termed IBD–IBS crossover. IBD–IBS crossover is typified by chronic visceral pain, which negatively impacts the quality of life, and is frequently referred to as a functional-organic overlap.^7, 8^ Factors that have been proposed to explain this overlap include psychosocial stress, genetics, altered microbiome, and aberrant epithelial immunology.^9^ This crossover though is poorly understood, and it is unclear whether IBD–IBS crossover is similar to the disease pattern of IBS or is a separate entity. When pain is present in quiescent IBD, the cause is incompletely understood, but it is considered that visceral hypersensitivity may exert an important effect.^10, 11^

Visceral sensitivity is usually evaluated by measuring rectal sensitivity to mechanical (manual or automated using a barostat), nutrient, chemical, thermal, or electrical stimuli, to discriminate between hyper, normo, and hyposensate populations. The intensity of pain using such techniques is most commonly measured using a self-report visual analog scale. Rectal hypersensitivity is well studied in IBS and is one of the leading hypotheses for the origin of symptoms in IBS. The basis for rectal hypersensitivity includes alterations both at the peripheral level such as altered barrier function and sensitized afferent nerves and at the central spinal dorsal horn and brain level leading to functional disturbances where physiological stimuli can lead to pain. There is a correlation between the degree of rectal hypersensitivity in IBS patients and symptom severity scores.^12^

Provocation tests suffer from significant heterogeneity as distension protocols and definitions for a painful stimulus vary from study to study, although recent international efforts have sought to improve standardization.^13^ Repeated exposure to experimental provocation stimuli can normalize rectal sensation probably due to habituation.^14^ However, mechanical stimulation is currently regarded as the most reliable instrument to assess rectal sensitivity.^15^

The primary aim of the study was to assess if pain thresholds to mechanical rectal stimulation are different in the IBD and healthy control population. Secondary aims were to assess if there was a difference between rectal sensitivity in active and quiescent disease.

## Materials and Methods

### Study Population and Study Design

The systematic review and meta-analyses were conducted according to the PRISMA recommendations and were registered with PROSPERO (Reference CRD42018095687).^16^ The search of the literature was performed using MEDLINE and EMBASE (1970 to June 2018). This was carried out using the set search strategies outlined in Table S1. There were no language restrictions. Eligibility criteria are given in Box 1. The bibliographies of all relevant studies and available meeting abstracts were screened to identify studies that were missed by the original search criteria. Senior authors were contacted to provide additional information where required. Articles were assessed independently by 2 reviewers using the predetermined eligibility criteria. Disagreements were resolved by consensus.

**Box 1. Eligibility Criteria**

Inclusion criteria:

- Diagnosis of ulcerative colitis or Crohn’s disease.
- Assessment of IBD and a healthy control population.
- Measurement of pain/discomfort thresholds using mechanical rectal distension.
- Prospective study.

Exclusion criteria:

- Testing pain/discomfort thresholds by means other than mechanical such as electrical.
- Retrospective studies due to the risk of repeat data.

### Data Extraction

The names of the first author, year of publication, location of study, IBD population size, control population size, IBD diagnosis, disease activity, and primary outcome data were recorded in means and standard deviations into an Excel Spreadsheet (Excel 2016; Microsoft).

The primary outcome was to determine if there was a difference in pain/discomfort threshold in IBD versus control populations. Secondary outcomes were to determine if the prevalence of rectal hypersensitivity differed in active versus quiescent IBD disease compared to healthy controls. Standard deviations were calculated according to the Cochrane Collaboration guidelines.^17^

### Quality Assessment and Risk of Bias

Two investigators performed a bias assessment independently for all studies included in the meta-analysis. Bias was scored in 4 areas using a modified checklist for case–control studies.^18, 19^ These areas were (1) blinding of assessors, (2) use of aged-matched controls, (3) use of gender-matched controls, and (4) controlling for other known factors that affect pain sensation.

### Data Analysis

Data were pooled for meta-analysis and a random effect model using the Hartung-Knapp-Sidik-Jonkman method was chosen. Heterogeneity was assessed using the *I*^2^ statistical test which gives values between 0% and 100%, with 0% representing no observed heterogeneity. Outcomes were assessed using Hedges’ g effect sizes and are reported with 95% confidence intervals (CIs). A prespecified secondary analysis was performed to determine if the effect size was modified in various subgroups. The statistical criterion was *P* < .05. Evidence of publication bias was assessed by using a funnel plot and Egger’s test. Propriety software was used to perform the meta-analysis and generate the plots (Comprehensive Meta-Analysis Version 2, Biostat, Version 2, and R Foundation for Statistical Computing).

## Results

### Search Results

**Figure 1.**
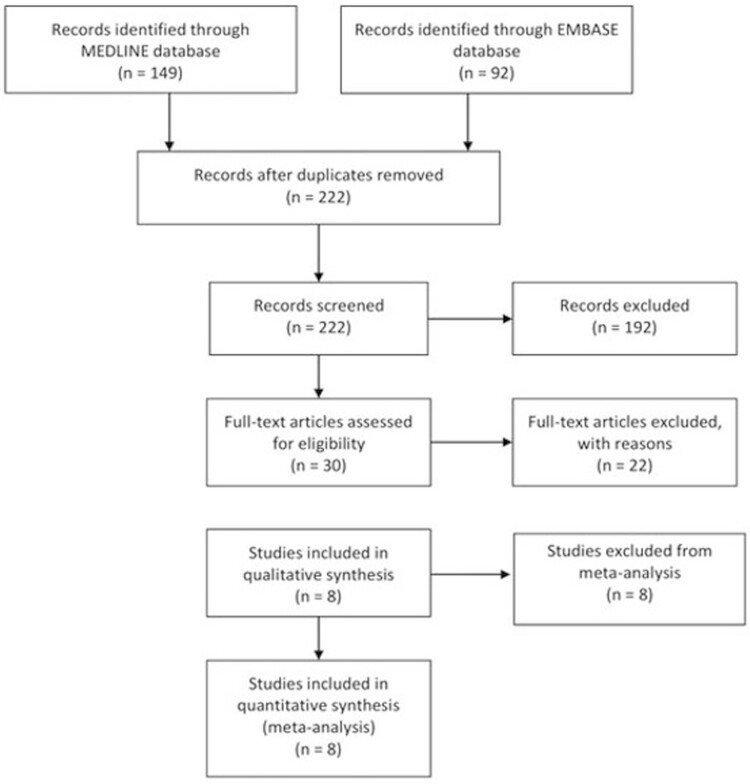
Flow diagram showing the studies included in the meta-analysis.

The search generated 222 citations of which 16 were classed as relevant and 8 met the inclusion criteria comprising 133 individuals with IBD and 99 healthy controls, 39 studies were rejected (Figure 1). The characteristics of the included studies including disease extent are given in Table S2. Twenty-nine of the participants had CD and 123 had UC. One study evaluated pediatric patients defined as being younger than 18 while the rest evaluated adults (10–77 years). Control ages were between 10 and 67 years. About 43% of IBD participants and 44% of the control population were female.

### Rectal Sensitivity in IBD

**Figure 2.**
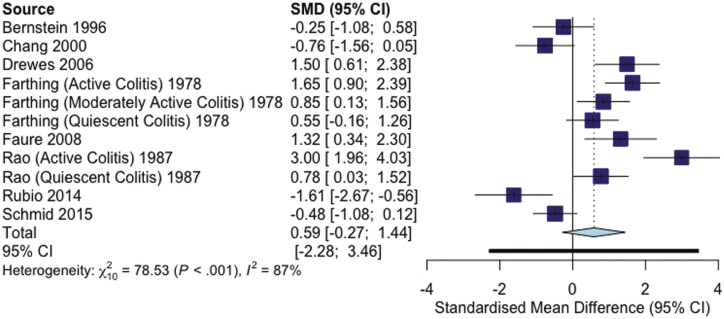
Forest plot of effect sizes for pain thresholds in IBD and healthy controls.

There was no significant difference in rectal pain thresholds in IBD patients compared to healthy controls with an effect size of 0.59 (95% CIs: −0.27 to 1.44; *P* = .16; *I*^2^ = 87.3%; Figure 2).

### Subgroup Analysis

All studies provided information about whether they were assessing IBD patients in either active or quiescent state. Three studies provided data on patients with active disease and 6 provided data on patients with quiescent disease. The active disease group included 50 people with active IBD compared to their control populations of 31. In the quiescent population, the 6 studies included 83 people with quiescent IBD and their control population of 68. Subgroup demographics are illustrated in Table S4. The active IBD group only covered UC whereas the quiescent subgroup also contained CD patients. Only one study in the quiescent IBD group included patients with chronic abdominal pain who may represent IBD–IBS crossover.^20^ This particular study did have a highly positive effect size.

**Figure 3.**
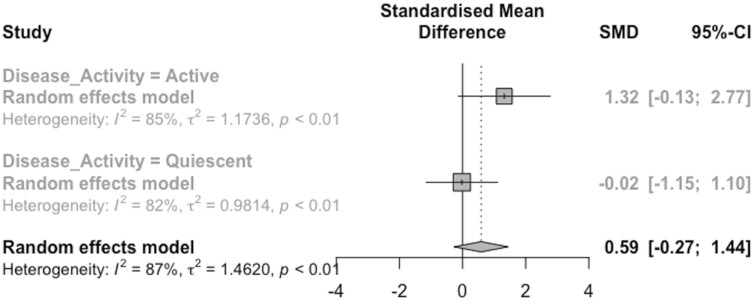
Forest plot for different disease activities.

A significant difference was observed between the groups (*P* = .048). The effect size in those with active disease was 1.32 (95% CIs: −0.13 to 2.77) compared to −0.024 (95% CIs: −1.15 to 1.10) in those with quiescent disease (Figure 3).

### Heterogeneity

Heterogeneity was very high as demonstrated by *I*^2^ = 87%, which is likely due to the differences in populations studied, definitions of active and quiescent disease, and rectal distention protocols. A number of statistical tests were performed to assess for heterogeneity including a Baujat plot which showed that 2 of the studies had a large effect of heterogeneity, but when these were removed heterogenicity remained high at 80% and the effect size was relatively unaffected at 0.55.

### IBD Study Quality Assessment

The quality of the studies included in the meta-analysis is summarized in Table S2. Of the studies included, 3 looked at CD with a variety of disease locations with some colonic disease. The other studies looked at UC with a variety of disease locations. All studies used a similar painful stimulus of mechanical rectal stimulation. All of the studies had small populations. The subject population studied were all middle- and young-aged adults except for a single pediatric study that contained only adolescent participants between 10 and 18.

The risk of bias within individual studies was scored out of 8, and the results are outlined in Table S3. All of the studies included in this trial were scored as being at high risk of bias in their protocols, but their participants were relatively well matched.

### Publication Bias

A funnel plot was calculated to look at the risk of publication bias, which is shown in Figure S5. An Egger’s test was performed to assess for asymmetry and did not reveal any funnel plot asymmetry.

## Discussion

This meta-analysis demonstrates that rectal hypersensitivity is not more common in IBD populations in comparison to healthy controls, but rectal sensitivity differs between patients and controls when disease activity is taken into account. The implication of these findings is that inflammation in active disease can lead to rectal hypersensitivity, possibly through a combination of peripheral and central mechanisms.

In our study, IBD per se was not associated with rectal hypersensitivity in comparison to healthy controls. This observation is likely due to the heterogeneity of the IBD population included in this study, with a predominance of those with quiescent disease without abdominal pain who did not demonstrate rectal hypersensitivity. This quiescent group is therefore unlikely to demonstrate rectal hypersensitivity because previous studies suggest a correlation between the degree of rectal hypersensitivity and symptom severity scores.^12^ Thus, it is not surprising that when the IBD population is taken as a whole, then we did not observe the presence of rectal hypersensitivity.

Hitherto, there has been considerable debate as to whether patients with quiescent IBD who complain of abdominal pain have concomitant IBS or mild ongoing inflammation that is not readily identifiable using current techniques.^21^ The situation is further confounded by reports of the existence of subtle inflammation or immune activation in patients with IBS where rectal hypersensitivity is thought to be present.^22, 23^ However, when rectal sensitivity was tested during active flares of the disease, a significantly greater effect size was noted in IBD patients compared to healthy controls, which is consistent with the sensitizing effect of inflammation on afferent neurons.^24^ This sensitizing effect of inflammation would lead to an increase in certain neurotrophic factors that are involved in gut nociception, such as nerve growth factor, and heightened expression of the transient receptor potential cation channel subfamily V member 1 (TRPV1) and the purinergic P2X3 receptor in the mucosa.^25–27^ After the inflammation subsides, it would be expected that these neurotrophic factors would return to near-normal levels which would lead to a reduction in abdominal pain and rectal hypersensitivity. However, it appears that in people with IBD–IBS overlap, a reduction in levels of these nociceptive channels particularly TRPV1 does not occur,^28, 29^ and their levels are significantly related to the abdominal pain score. This would indicate that the predominant mechanism of abdominal pain in quiescent disease is likely related to peripheral sensitization of nociceptive receptors despite the healing of inflammation itself.

Hypervigilance to stimuli is known to play a role in the development of pain and rectal sensitivity. Hypervigilance is common in experimental settings as the stimulation represents a “threat” to the participant, but repeated exposure to the stimulus will lead to habitation. Abdominal pain-related fear learning and memory processes are altered, which may contribute to central pain amplification and hypervigilance which may be enhanced in those with comorbid anxiety and depression.^30^ Experimentally induced negative emotions during painful rectal distension even in healthy volunteers can lead to increased brain activity in the left thalamus and right dorsal posterior cingulate gyrus.^31^ Hypervigilance is known to be associated with anxiety and depression; however, none of the studies included in this meta-analysis were corrected for anxiety and depression and the majority did not measure anxiety and depression scores. Currently, the role of hypervigilance in IBD patients with and without rectal hypersensitivity is unknown and requires further study.

People with quiescent IBD who do not experience chronic pain are known to have activation of their anti-nociceptive pathways^32^ which includes descending inhibitory control from the brain which can reduce peripheral sensitization. However, it has been shown that descending inhibition is deficient in IBS patients with rectal hypersensitivity.^5^ It is thus possible that there are differences in the activity of descending inhibitory pathway between IBS and IBD patients in remission such that while in IBS these pathways are less active they may be more active in quiescent IBD patients who do not suffer from chronic abdominal pain. Further research is required to confirm these hypotheses.

This study has several limitations. A major issue is to do with the very high level of heterogenicity seen. There are likely to be multiple factors affecting the level of heterogenicity as already described above. Pain itself is a highly variable subjective experience that will inevitably lead to increased heterogenicity.^10^ We corrected for the difference in study methodology by only evaluating studies that assessed rectal as opposed to colonic sensation. All of the studies included took place in tertiary care settings, so our data may represent a more severe phenotype than what is seen in other settings. A comparison between UC and CD was not attempted as the data were too heterogeneous and the number of eligible studies was small.

As previously mentioned, only a single article dealt with quiescent IBD patients with abdominal pain, that is, patients who may represent IBD–IBS crossover; further research is required looking to compare IBD–IBS crossover patients to quiescent IBD patients without pain and see if rectal stimulation-induced pain thresholds are different. Similarly, IBD–IBS crossover patients should be compared to IBS patients to see if they represent a similar phenotype. This would be highly clinically useful as it would show whether clinicians should be regarding IBD–IBS crossover as a subtype of IBS that could be treated accordingly or whether these are entirely different disease entities. Further studies should also look at correcting for anxiety and depression between the IBD–IBS population and the control population as these play significant roles in pain perception.

This meta-analysis indicates that rectal hypersensitivity is associated with IBD in the presence of active disease but not in the presence of quiescent disease in patients without chronic abdominal pain. Rectal hypersensitivity is therefore not a routinely seen feature in quiescent IBD but may still play a role in those who are suffering from IBS–IBD crossover.

## Supplementary Material

otab041_suppl_Supplementary_Materials

## Data Availability

No new data were created or analyzed in the publication of this work. All original data are available from referenced work.
