# Supplementary material for: Rectal Hypersensitivity in Inflammatory Bowel Disease: A Systematic Review and Meta-analysis
Source: Crohns Colitis 360. 2021 Jun 23;3(3):otab041. doi: 10.1093/crocol/otab041 (PMC9802320; doi:10.1093/crocol/otab041)
Supplement: otab041_suppl_Supplementary_Materials [file otab041_suppl_supplementary_materials.docx]

# supplementatry material

| **Pubmed**   1. Inflammatory Bowel Disease (MeSH) OR Inflammatory Bowel Disease 2. Ulcerative Colitis (MeSH) OR Ulcerative Colitis 3. Crohns Disease (MeSH) OR Crohns Disease 4. 1 OR 2 OR 3 5. Balloon distension OR Rectal distension OR Barostat OR Electrical Stimulation 6. 4 AND 5 7. 6 Filters: Humans 8. 7 Filters: Review 9. 7 NOT 8 | **EMBASE**   1. ‘crohn disease’/exp OR ‘crohn disease’ OR ‘ulcerative colitis’/exp OR ‘ulcerative colitis’ OR ‘inflammatory bowel disease’/exp OR ‘inflammatory bowel disease’ 2. ‘rectal distension’ OR ‘barostat’ OR ‘visceral hypersensitivity’ OR ‘electrostimulation’ 3. 1 AND 2 4. 3 limit to article, article in press, conference abstract, conference paper and human |
| --- | --- |

*Supplementary* *Material Table 1 - Search criteria used for Medline and Embase databases.*

**CHARACTERISTICS OF IBD STUDIES**

| **Study** | **IBD Size** | **Control Size** | **Disease Type and Location** | **Disease Activity** | **Excluded IBS type symptoms** | **Location of study** |
| --- | --- | --- | --- | --- | --- | --- |
| **Bernstein, 1996** | 12 | 9 | CD – Ileal disease | Quiescent | No current abdominal pain | United States |
| **Chang, 2000** | 11 | 13 | UC - 8 left sided, 3 pancolitis | Quiescent | Excluded if met Rome criteria | United States |
| **Drewes, 2006** | 9 | 17 | UC – 7 proctitis, 2 left sided | Active | Active disease | Denmark |
| **Farthing, 1978** | 34 | 15 | UC – 20 pancolitis, 14 left sided | 23 Active  11 Quiescent | No mention of IBS or symptoms | United Kingdom |
| **Faure, 2008** | 8 | 10 | Paedatric CD – 2 Colonic, 4 ileal, 2 small bowel | Quiescent | All complained of chronic abdominal pain | Canada |
| **Rao, 1987** | 35 | 12 | UC | 18 Active  17 Quiescent | 1 quiescent disease had abdominal pain | United Kingdom |
| **Rubio, 2014** | 9 | 8 | CD – 6 small bowel, 3 colonic | Quiescent | Excluded if met Rome III criteria | France |
| **Schmid, 2015** | 15 | 15 | UC | Quiescent | Excluded if abdominal pain | Germany |

*Supplementary* *Material Table 2 – Characteristics of the IBD studies.*

**QUALITY ASSESSMENT OF IBD STUDIES**

| **Study, Year** | **Blinding of outcome assessors** | **Matching for age** | **Matching for gender** | **Discrepancies between IBD and control** | **Total Bias Score** |
| --- | --- | --- | --- | --- | --- |
| Bernstein, 1996 | 2 | 0 | 0 | 2 | 4 |
| Chang, 2000 | 2 | 2 | 0 | 0 | 4 |
| Drewes, 2006 | 2 | 0 | 2 | 2 | 6 |
| Farthing, 1978 | 2 | 1 | 0 | 2 | 5 |
| Faure, 2008 | 2 | 0 | 2 | 0 | 4 |
| Rao, 1987 | 2 | 2 | 0 | 1 | 5 |
| Rubio, 2014 | 2 | 1 | 1 | 0 | 4 |
| Schmid, 2015 | 2 | 2 | 0 | 0 | 4 |

Supplementary Material Table 3 – Quality Assessment of IBD Studies

# Demographics of subgroup analysis

|  | Active IBD | Quiescent IBD |
| --- | --- | --- |
| Minimum Age | 23 | 10 |
| Maximum Age | 77 | 74 |
| Mean Age | 42.3 | 40.1 |
| Female | 27 | 39 |
| Male | 23 | 44 |

Supplementary Material Table 4 – Demographics of Subgroup Analysis


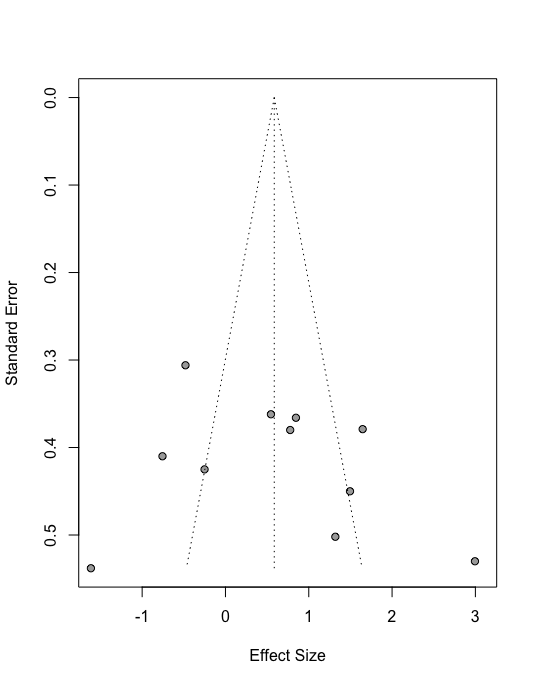


Supplementary Material Figure 5 – Funnel Plot to show for publication bias
